# Task-related modulation of effective connectivity during perceptual decision making: dissociation between dorsal and ventral prefrontal cortex

**DOI:** 10.3389/fnhum.2013.00365

**Published:** 2013-07-15

**Authors:** Rei Akaishi, Naoko Ueda, Katsuyuki Sakai

**Affiliations:** ^1^Department of Cognitive Neuroscience, Graduate School of Medicine, The University of TokyoTokyo, Japan; ^2^Department of Experimental Psychology, University of OxfordOxford, UK

**Keywords:** transcranial magnetic stimulation, electroencephalography, frontal eye field, ventral prefrontal cortex, perceptual decision making, accumulation, selection

## Abstract

The dorsal and ventral parts of the lateral prefrontal cortex have been thought to play distinct roles in decision making. Although its dorsal part such as the frontal eye field (FEF) is shown to play roles in accumulation of sensory information during perceptual decision making, the role of the ventral prefrontal cortex (PFv) is not well-documented. Previous studies have suggested that the PFv is involved in selective attention to the task-relevant information and is associated with accuracy of the behavioral performance. It is unknown, however, whether the accumulation and selection processes are anatomically dissociated between the FEF and PFv. Here we show that, by using concurrent TMS and EEG recording, the short-latency (20–40 ms) TMS-evoked potentials after stimulation of the FEF change as a function of the time to behavioral response, whereas those after stimulation of the PFv change depending on whether the response is correct or not. The potentials after stimulation of either region did not show significant interaction between time to response and performance accuracy, suggesting dissociation between the processes subserved by the FEF and PFv networks. The results are consistent with the idea that the network involving the FEF plays a role in information accumulation, whereas the network involving the PFv plays a role in selecting task relevant information. In addition, stimulation of the FEF and PFv induced activation in common regions in the dorsolateral and medial frontal cortices, suggesting convergence of information processed in the two regions. Taken together, the results suggest dissociation between the FEF and PFv networks for their computational roles in perceptual decision making. The study also highlights the advantage of TMS-EEG technique in investigating the computational processes subserved by the neural network in the human brain with a high temporal resolution.

## Introduction

Perceptual decision making is understood as a process of accumulating task-relevant sensory information toward a decision threshold (Gold and Shadlen, 2007). It has been shown that neurons in the frontal eye field (FEF) as well as lateral intraparietal region of monkeys show build-up of activity after presentation of a noisy sensory stimulus until the behavioral response. These activity patterns are taken to reflect the information accumulation process. However, in imaging studies of humans, not only the FEF but also the ventral prefrontal cortex (PFv) around the posterior portion of the inferior frontal sulcus are shown to be active in discrimination of sensory stimuli (Binder et al., 2004; Pessoa and Padmala, 2005; Ploran et al., 2007; Thielscher and Pessoa, 2007; Kayser et al., 2010; Liu and Pleskac, 2011). It has been proposed that the PFv plays a role in allocation of attentional resources to maintain accuracy of decision making, possibly by sending selection signals to sensory areas to collect choice-relevant information (Corbetta and Shulman, 2002; Heekeren et al., 2008).

A crucial question here is whether the selection process in the PFv can be distinguished from information accumulation process in the FEF. It also remains open how the processes of information accumulation and selection interact in the decision network. To answer these questions, we used concurrent transcranial magnetic stimulation (TMS) and electroencephalography (EEG) recording (TMS-EEG) (Komssi and Kähkönen, 2006; Driver et al., 2009; Siebner et al., 2009; Miniussi and Thut, 2010; Reithler et al., 2011; Daskalakis et al., 2012; Rogasch and Fitzgerald, 2013), and examined neural network connectivity involving the FEF and PFv during perceptual decision making. A single pulse of TMS over a given cortical region induces spread of neural impulses from the stimulated region toward the anatomically connected regions (Ilmoniemi et al., 1997), and the pattern of neural impulse transmission changes depending on the state of local and inter-regional neural network downstream to the stimulated region (Massimini et al., 2005; Esser et al., 2009; Morishima et al., 2009; Akaishi et al., 2010). We reasoned that we can make inference about the cognitive or computational processes subserved by the network involving the stimulated region by analyzing experimental or behavioral factors that modulate the scalp distribution of TMS-evoked potentials in EEG (TMS-EPs). Because of the causal relationship between the stimulation and EEG responses, this technique of TMS-EEG reveals how the stimulated region interacts with other regions of the network. In the present study, we analyzed the TMS-EPs after stimulation of the FEF and PFv, and show double dissociation between the FEF and PFv for their roles in information accumulation and selection. We also show that the functional networks of the FEF and PFv overlap in the medial and dorsolateral prefrontal cortex.

## Materials and methods

### Subjects

Twenty normal human subjects participated in the experiment with FEF stimulation (8 females; age: 20–42), and 13 in the experiment with PFv stimulation (6 females; age: 21–46). Written informed consents were obtained from all the subjects prior to the experiments. The study was approved by the ethics committee of the Graduate School of Medicine, the University of Tokyo.

### Behavioral paradigm

**Figure 1:**
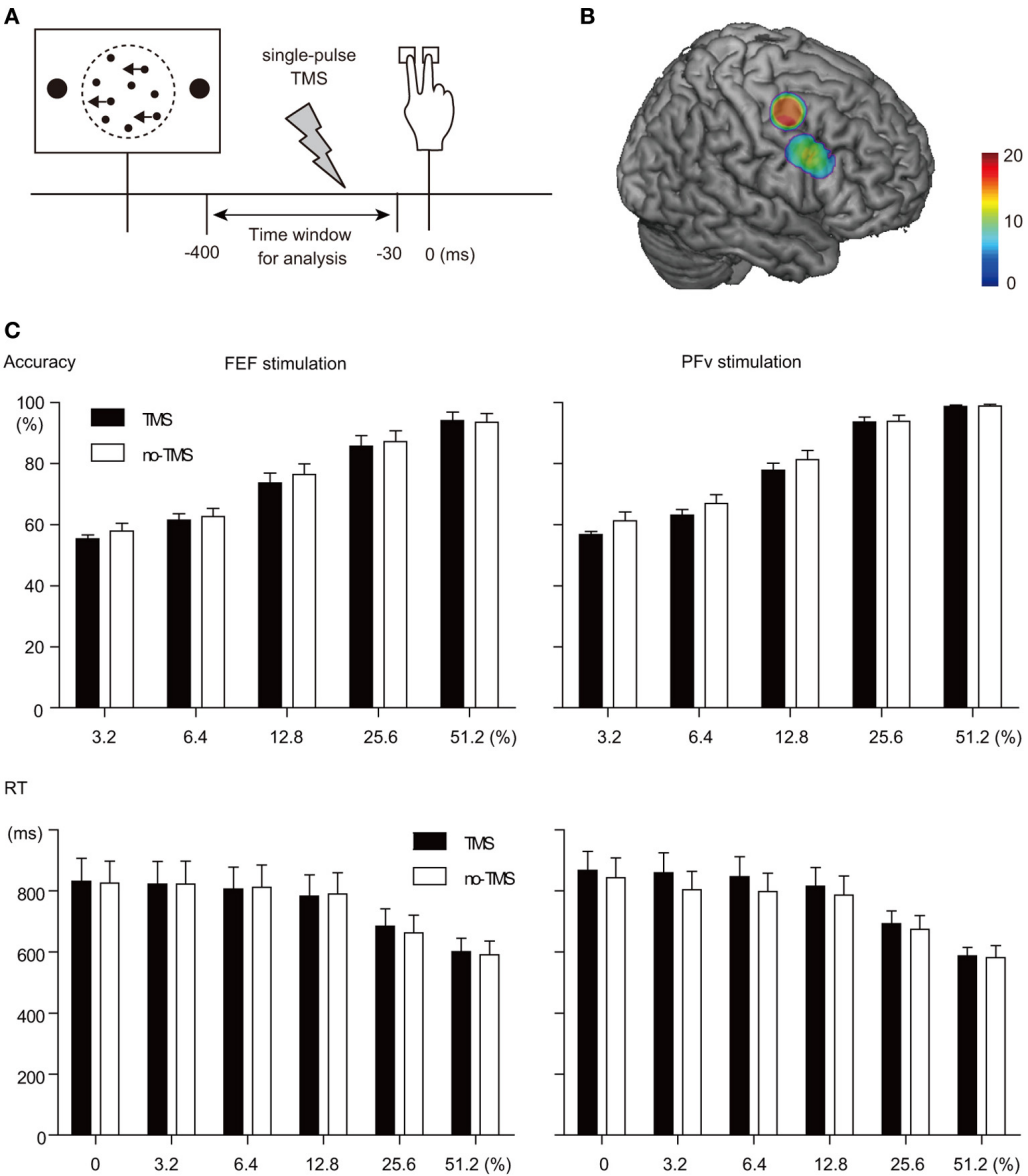
**TMS manipulation experiment. (A)** Behavioral paradigm. A single-pulse TMS was given at variable timing between visual stimulus onset and behavioral response. Trials in which TMS was given within the time window between 30 and 400 ms before behavioral response were analyzed. **(B)** Stimulation sites rendered on a template MNI brain. Dorsal and ventral clusters indicate FEF and PFv stimulation sites, respectively. Color bar indicates the number of overlapped subjects. Note that the number of subjects was 20 and 13 for FEF and PFv stimulation, respectively. **(C)** TMS effects on behavior. Accuracy (top) and RT (bottom) for each motion coherence level (abscissa) are shown separately for FEF (left) and PFv stimulation (right).

Subjects performed a reaction time version of the two-direction motion discrimination task with manual response (Figure 1A). The stimulus was a set of white dots (123.1 cd/m^2^, size: 0.06° of visual angle, mean density of 49.6 dots/deg^2 *^s) displayed within an invisible circular aperture (5° in diameter) at the center of a dark background (1.8 cd/m^2^). The refresh rate of the monitor was 60 Hz. A subset of dots was offset from their original position every 50 ms to create apparent motion to the left or right at 5.0°/s and the remaining dots were moved to random locations. The percentage of the dots that were moving in the same direction was manipulated at 0, 3.2, 6.4, 12.8, 25.6, and 51.2%. Trials with 0% motion coherence were excluded from the analysis of TMS-EPs because the accuracy of performance cannot be examined. The direction of motion and motion coherence level were pseudo-randomized within an experimental session, such that the same number of trials for left- and right-ward motion for each of motion coherence level were presented. Subjects were asked to indicate the perceived direction of coherent dot motion by pressing a button with the index or middle finger of the right hand, as accurate and quickly as possible. The random dot motion pattern disappeared when subjects made button press or when 2 s elapsed without button press. The response-stimulus interval was varied from 1320 to 1590 ms, and 120 trials x 12 sessions were performed by the subject in the TMS-EEG session. Before the experiment, the subject performed two practice sessions, 120 trials for each, to achieve stable performance in the TMS-EEG session.

### Measurement of TMS-EPs

In the concurrent TMS-EEG session, we gave a single-pulse TMS on half of the trials in each session of 120 trials using a figure-eight shaped coil (70 mm diameter) and MagStim 200 stimulator (MagStim, UK), while TMS-evoked scalp-recorded potentials were recorded using EEG. Position of the TMS coil was adjusted using Brainsight (Rogue Research, UK) based on the structural MRI of the individual subjects' brain. The FEF was determined as the region just below the junction between the superior frontal sulcus and precentral gyrus (Paus, 1996; Blanke et al., 2000; Lobel et al., 2001; Koyama et al., 2004; Grosbras et al., 2005). The PFv was determined as the region located just anterior to the junction of the posterior end of the inferior frontal sulcus and the inferior limb of the precentral sulcus, which has been shown to be active during perceptual decision making in the previous studies (Heekeren et al., 2006; Kayser et al., 2010; Liu and Pleskac, 2011) (Figure 1B). Mean coordinate for the FEF stimulation was (38, −3, 50), and that for the PFv stimulation was (53, 13, 30). According to the probabilistic cytoarchitectonic atlas (SPM Anatomy toolbox), the FEF in the present study corresponds to the border between Brodmann's area (BA) 6 and 8, whereas the PFv corresponds to the border between BA44 and 45. For FEF stimulation, TMS coil was oriented 45° from the middle line with its handle pointed posteriorly. For PFv stimulation, TMS coil was oriented parallel to the middle line of the head with its handle pointed posteriorly. The TMS intensity was 35% of the maximum stimulator output, which did not exceed the active motor threshold: The TMS intensity was 69.4% (range: 53–88) and 73.7% (55–92) of the active motor threshold for the FEF and PFv stimulation, respectively. In contrast to other studies of TMS, the low-intensity TMS was used as a means to probe the state of the neural network, rather than as a means to manipulate the underlying neural processes.

TMS was delivered at a variable timing between the stimulus onset and behavioral response. For each subject, we first determined the average RT for each motion coherence level based on the behavioral data in the practice sessions (240 trials in all). During the TMS-EEG experiment, we gave TMS at a variable timing relative to the stimulus onset, with the latest timing determined based on the estimated RT. The estimation of the RT was updated for each experimental session so as to take into account the change in behavior during the experiment. When the response key was pressed earlier than the preprogrammed timing of TMS, the TMS trigger pulse was aborted and no TMS was given. After the experiment, trials were sorted *post-hoc* depending on the time relative to the behavioral response (Figure 1A). This was to examine the change in the TMS-EPs according to the relative time to behavioral response.

Throughout the experimental session, we recorded EEG with 60 electrodes placed according to an extended 10/20 system using a TMS-compatible amplifier (BrainAmp, Brain Products, Germany). EEG signals were referenced to the mean of all electrodes, and were low-pass filtered at 1000 Hz, DC-corrected, and sampled at 2500 Hz with 16 bit resolution. Impedance of each electrode was kept below 5 kΩ for all experiments. Eye movements were also recorded by tracking the pupillary position of the left eye at a sampling rate of 60 Hz using ViewPoint eye tracker (Arrington Research, AZ).

EEG data were preprocessed with BrainVision Analyzer (BrainProducts, Germany) and custom programs on MATLAB (Mathworks, MA). We then used the SPM8 (http://www.fil.ion.ucl.ac.uk/spm/) for statistical analysis and data visualization. Artifacts due to TMS were observed on channels around the stimulation site but in the majority of trials they disappeared within 8 ms of the stimulation. Trials with prolonged TMS artifacts were removed: We rejected trials with amplitude larger than 50 μV relative to the baseline during the time window of 8–40 ms after TMS. Trials with muscle activity, blinking artifacts and eye movements were also removed. The mean rejection rate was 31.6% (24.0–39.7) for FEF stimulation and 31.7% (25.1–41.4) for PFv stimulation. The pattern of TMS-induced artifacts did not show a time-dependent change: Trials with large artifacts due to TMS appeared randomly throughout the experimental sessions. This may indicate a subtle change in the coil position, and sometimes the coil may have contacted directly with the electrode leads, causing the artifacts. But the stimulation site monitored by the navigator system was localized within a region of 5 mm in diameter and thus in terms of the stimulated cortical region, the position of the TMS coil was considered to be maintained stably.

After rejection of trials with artifacts, the EEG waveforms on TMS-trials were aligned at the onset of TMS, and were baseline-corrected based on the data within the 4-ms pre-TMS period. In the present study, TMS was given at a variable timing during perceptual decision making, and EEG during the pre-TMS period is not flat and differs across trials even within the same condition. We thus chose to use a time-window of 4 ms as a reference to correct the baseline in order to align the amplitude of EEG at the time of TMS at the zero point. In other words, this duration was arbitrarily chosen to reduce the noise. The problem of using such an extremely short period for baseline correction is the contribution of the phase of oscillatory EEG activity at the time of TMS, and we need to obtain a large number of trials for averaging to cancel out the effect.

### Analysis of TMS-EPs

We focused on two experimental factors that would modulate the TMS-EPs, which are the TMS timing relative to the behavioral response (Time-to-Response) and accuracy of behavioral response (Accuracy). It has been shown that in single unit recording studies in monkeys, activity of neurons in the FEF increases gradually from 200 ms from the stimulus onset until the time of behavioral response (Gold and Shadlen, 2007). Based on these findings, we examined changes in the TMS-EPs according to the TMS timing relative to the behavioral response: In other words, we examined changes in the effective connectivity associated with the amount of accumulated sensory information. The prediction is that the TMS-EPs after FEF stimulation are modulated by the factor of Time-to-Response. By contrast, the previous findings suggest the role of PFv in attentional selection processes (Corbetta and Shulman, 2002; Heekeren et al., 2008). The efficiency in selection of task-relevant information is thought to be associated with accuracy of behavioral performance (Pelli, 1985; Shadlen et al., 1996). Based on these previous studies, we expected that the TMS-EPs after PFv stimulation are modulated by the factor of Accuracy.

During the TMS-EEG experiment, we gave TMS at a variable timing relative to the stimulus onset. After the experiment, trials were sorted *post-hoc* depending on the time relative to the behavioral response. The TMS-EPs data were categorized according to whether the TMS was given early (400–130 ms before response) or late (130–30 ms before response) during the decision process (factor of Time-to-Response, early or late). This categorization of the time windows for analysis was determined based on the time course of firing of FEF neurons obtained from monkeys performing the same motion discrimination task for random dot patterns. It has been shown that when the neuronal firing patterns are aligned to behavioral response, the build-up of FEF neuronal firing starts from around 400 ms before the onset of saccade response and takes a peak value at roughly 30 ms before the saccade response (Ding and Gold, 2012). Although a manual response paradigm was used in the present study instead of a saccade paradigm used in monkey studies, we consider that the FEF neurons show a similar build-up of activity in the manual response paradigm. In fact, a previous imaging study suggests a similar build-up of activity in the FEF between saccade and manual response paradigms (Liu and Pleskac, 2011). Thus, the time window of 30–400 ms before response can be taken to correspond to the build-up phase of neuronal firing, which has been associated with accumulation of decision-related sensory information. We further considered that the FEF neuronal activity in a manual response paradigm may reach a plateau at about 100 ms before the response: Compared to a saccade paradigm, the reaction time in the manual response paradigm is longer by about 100 ms. Thus, the comparison between Early (130–400 ms before response) and Late (30–130 ms before response) epochs can be taken to reflect the difference between the neuronal build-up phase and plateau phase. TMS was given more often during the later period during the decision so as to roughly equate the number of trials between Early and Late. Trials in which TMS was given outside these time windows were excluded from the analysis. The limitation is that these time windows of analysis are based on the single unit firing data obtained from monkeys, which may not be directly applicable to human studies.

We also categorized the TMS-EPs data according to the accuracy of choice response given the sensory information on that trial (factor of Accuracy, correct or error). For the TMS-EPs data thus arranged in a 2-by-2 factorial design, we tested the main effects of Time-to-Response and Accuracy, as well as the interaction between the two factors. We focused on the TMS-EPs within the time window of 8–40 ms after TMS in order to avoid the period that contains artifacts due to the TMS pulse. We also restricted the analysis within the interval of 40 ms after TMS because we were interested in initial spreading patterns of the neural impulse induced by the TMS, which most likely reflects direct impulse transmission from the stimulated region. Using SPM8 for EEG (http://www.fil.ion.ucl.ac.uk/spm/), the TMS-EP data were constructed in a three-dimensional space (*x* and *y* for space, and *z* for time), and the smoothness of the data across space and time was estimated to calculate effective degrees of freedom. To ensure smoothness assumption of the random field theory, we used Gaussian spatial filter with full width half maximum (FWHM) of 48 mm and Gaussian temporal filter with FWHM of 8 ms. Average TMS-EPs for each trial type were calculated for each subject, and the main effects of Accuracy and Time-to-Response and their interaction were tested across subjects. We used a statistical threshold of *p* < 0.05 corrected for multiple comparisons in spatial as well as in time domains.

The effects of the Time-to-Response and Accuracy were also tested on EEG data on no-TMS trials. This was to examine whether or not the effect of Time-to-Response or Accuracy that could be observed on TMS-EPs is due to modulations in the baseline EEG pattern. For this purpose, we extracted epochs of no-TMS trials that match with the time window of analysis for TMS trials. For each TMS trial, we searched a matched no-TMS trial in which a visual stimulus with the same motion coherence was presented as in that particular TMS trial and also in which the RT was within the range of 100 ms relative to the RT of the TMS trial. The TMS trial was excluded from the analysis when we failed to find a matched no-TMS trial. Thus the TMS and no-TMS trials are matched roughly in a pair-wise manner in terms of the motion coherence level of the stimulus and response time. We then extracted an epoch for analysis from the matched no-TMS trial thus selected; the epoch was determined as a 32-ms period with the same time-to-response as the time window of 8–40 ms after TMS in the matched TMS trial. The conventional approach is to subtract the EEG waveforms on no-TMS trials from those on TMS trials so that we can examine the potentials induced by the TMS. In the present study, however, the TMS timing is varied across trials. The time window for ERP analysis is also varied. It can be problematic to subtract waveforms obtained from variable epochs of no-TMS trials in terms of their timing from those obtained from variable epochs of TMS trials. We tried to extract an epoch for no-TMS trial that corresponds to the epoch for a given TMS trial in a pair-wise manner, but the timings of the epochs were not matched exactly. We therefore applied Two-Way ANOVA (with factors of TMS timing and Accuracy) separately for TMS and no-TMS trials.

In a separate model, we also tested the effect of the timing of TMS relative to the visual stimulus onset (factor of Time-from-Stimulus, categorized as early or late depending on whether the TMS was given between 170 and 470 ms or later than 470 ms after stimulus onset). These time windows roughly correspond to the build-up phase of FEF neuronal firing when the neuronal firing is aligned to visual stimulus onset (Ding and Gold, 2012). The division between the Early and Late time windows at 470 ms after visual stimulus onset was to equate the number of trials between Early and Late. Trials analyzed for the effect of Time-to-Response and those analyzed for the effect of Time-to-Stimulus are identical. Of note is that trials categorized as Early in terms of Time-to-Response do not necessarily corresponds to trials categorized as Early in terms of Time-from-Stimulus. The same is true for Late trials.

We also tested the effect of motion coherence level of the stimuli, which was categorized as low (3.2 and 6.4%) or high (12.8, 25.6, and 51.2%). This is to examine if the effect of Accuracy is confounded by the motion coherence (i.e., stimulus strength). In Table 1, we report the number of trials composing Early and Late TMS and Correct and Error trials, separately for low and high motion coherence. As shown in the table, the number of trials differs greatly between Correct and Error for high coherence motion trials, but there were at least 25 error trials in high coherence condition, which were enough, though not ideal, for the analysis.

**Table 1:** **Number of trials used for TMS-EP analysis (mean and range)**.

	**Low coherence**	**High coherence**
**FEF STIMULATION**		
Early	149 (101–173)	206 (136–271)
Late	138 (95–197)	229 (171–263)
Correct	213 (195–264)	386 (320–412)
Error	74 (49–105)	49 (25–65)
**PFv STIMULATION**		
Early	158 (119–174)	211 (150–271)
Late	143 (106–188)	208 (176–259)
Correct	219 (195–264)	356 (301–417)
Error	82 (43–129)	63 (27–93)

Motion coherence of low coherence trials: 3.2 and 6.4%.

Motion coherence of high coherence trials: 12.8, 25.6, and 51.2%.

### Source estimation of signal transmission from the FEF and PFv

We next identified regions that receive signals from the FEF and PFv using cortical source density analysis based on scalp distribution of TMS-EPs. TMS-EPs were averaged across all trials for each subject and for each stimulation site. We then used sLORETA (Pascual-Marqui, 2002) and estimated the cortical distribution of current source density (CSD) that accounts for the scalp distribution of TMS-EPs during the time window of 20–40 ms after the TMS. This time window was chosen based on the previous studies showing TMS-induced activation in distant regions (Ilmoniemi et al., 1997; Massimini et al., 2005; Morishima et al., 2009; Akaishi et al., 2010) and also based on the results of present study showing significant effect of Time-to-Response and Accuracy on TMS-EPs within this time window. Computations of CSD were performed in a realistic head model, using a template brain of the Montreal Neurological Institute (MNI152), with the three-dimensional solution space restricted to cortical gray matter. The intracerebral volume was partitioned in 6239 voxels at 5 mm spatial resolution. The logarithmically-transformed CSD values for each voxel of the MNI space were compared against zero using one-sample *t*-test. We used a non-parametric permutation test with a threshold of *p* < 0.05 corrected for multiple voxels based on 5000 randomizations (one-tailed).

## Results

### TMS effect on behavior

Stimulation of each network had only minimal effects on behavior. Accuracy of task performance decreased slightly after the TMS (mean across subjects: 1.5 and 2.4% decrease for FEF and PFv stimulation, respectively), but the effect did not differ significantly between the two stimulation sites and across motion coherence levels [Three-Way ANOVA on accuracy, Greenhouse-Geisser correction: Main effect of TMS: *F*_(1, 26)_ = 11.4, *p* = 0.002; interaction between TMS and stimulation site: *F*_(1, 26)_ = 0.62; *p* = 0.44; interaction between TMS, stimulation site and coherence: *F*_(2.447, 63.6)_ = 0.30; *p* = 0.78, Figure 1C top]. Response time (RT) of the performance was not affected by the stimulation for either site, and the interaction with motion coherence level was not significant [Three-Way ANOVA on RT, Greenhouse-Geisser correction: Main effect of TMS: *F*_(1, 26)_ = 0.003, *p* = 0.96; interaction between TMS and stimulation site: *F*_(1, 26)_ = 0.83; *p* = 0.37; interaction between TMS, stimulation site and coherence: *F*_(3.34, 86.9)_ = 1.11; *p* = 0.35, Figure 1C bottom].

### Modulation of TMS-EPs

We examined the change in the TMS-EPs according to the TMS timing relative to the behavioral response. FEF neuronal activity has been shown to build up during perceptual decision making and reach a plateau just before the behavioral response (Kim and Shadlen, 1999; Ding and Gold, 2012). Based on the finding, we predicted that the TMS-EPs after FEF stimulation are modulated by how close TMS was at the time of behavioral response (Early or Late). By contrast, the previous findings suggest the role of PFv in attentional selection processes (Corbetta and Shulman, 2002; Heekeren et al., 2008). The efficiency in selection of task-relevant information is thought to be associated with accuracy of behavioral performance (Pelli, 1985; Shadlen et al., 1996). Based on these previous studies, we expected that the TMS-EPs after PFv stimulation are modulated by whether the subject select action based on the task relevant sensory information (Correct or Error).

**Figure 2:**
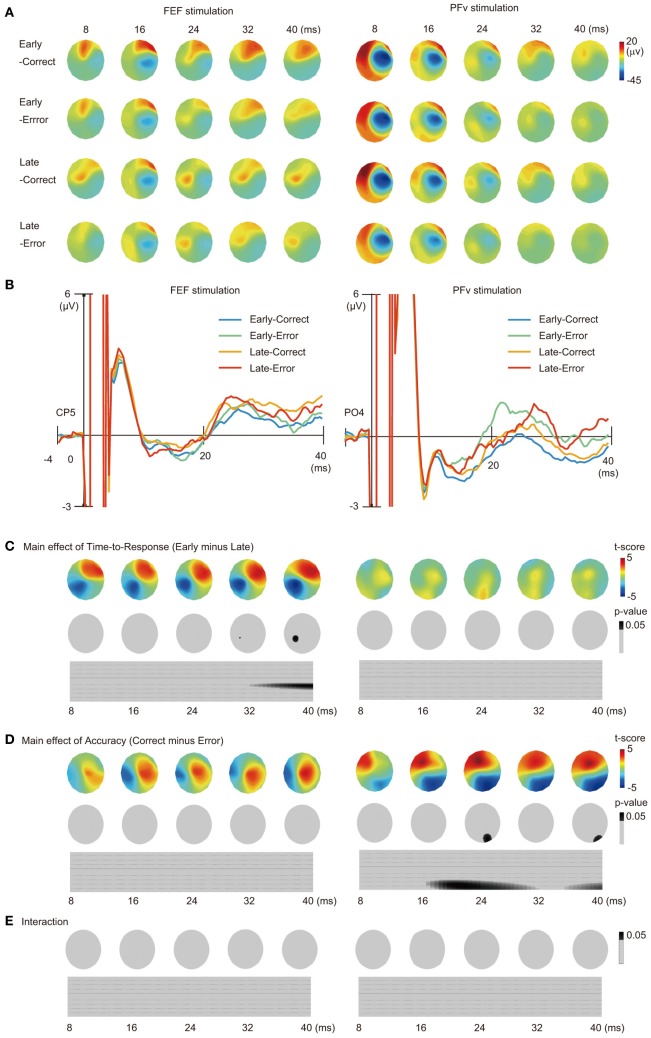
**TMS-EEG experiment. (A)** Scalp patterns of TMS-EPs shown in time bins of 8 ms after TMS. Data in which TMS was given early or late during the decision process and data in which subjects made correct or erroneous response are shown separately. FEF (left) and PFv stimulation (right). **(B)** Waveform of TMS-EPs from CP5 electrode in FEF stimulation (left) and that from PO4 electrode in PFv stimulation (right). Time bins in which there was a significant main effect of Time-to-Response factor (Early vs. Late) and those in which there was a significant main effect of Accuracy factor (Correct vs. Error) are indicated at the bottom of the trace by red tics. **(C)** Main effect of Time to Response (contrast: Early vs. Late) on TMS-evoked potentials. Scalp distribution of t-scores (upper row) and *p*-values (middle row, threshold: *p* = 0.05, corrected for family-wise error). Bottom row indicates 2-D plot of the continuous time series of *p*-values (abscissa: time from TMS; ordinate: electrode position with left anterior to right posterior electrode shown from top to bottom). Spatial and temporal windows with significant effects are indicated in black. **(D)** Main effect of Accuracy (contrast: Correct vs. Error). Same format as in **(C)**. **(E)** Interaction between Accuracy and Time-to-Response. Only the *p*-value maps are shown.

TMS on the FEF produced positive potentials in frontal electrodes and negative potentials in right temporo-parietal electrodes, which after 20 ms of TMS evolved into a pattern of left centro-parietal positive potentials (Figure 2A left). By contrast, TMS on PFv produced positive potentials in the left frontocentral electrodes and negative potentials in the right central electrodes, which after 20 ms evolved into a pattern of frontal positive potentials (Figure 2A right). As for the data at an electrode level, for FEF stimulation there was a larger positive deflection of the TMS-EP recorded from the CP5 electrode for Late than for Early trials (Figure 2B left). For PFv stimulation by contrast, there was a larger positive potential at the PO4 electrode for Error than for Correct trials (Figure 2B right).

To overcome the problem of multiple comparisons across electrode space and time, we conducted statistical analysis that took into account the multiple comparisons based on random field theory and smoothness estimate of our own data (in terms of both space and time). We found that TMS-EPs after FEF stimulation were significantly modulated by Time-to-Response especially in the left centro-parietal region, but not by Accuracy, whereas TMS-EPs after PFv stimulation were significantly modulated by Accuracy especially in the right parieto-occipital region, but not by Time-to-Response (*p* < 0.05. corrected for multiple comparisons across space and time; Figures 2C,D. Significant effects were observed within the time window of 20–40 ms after the TMS. Importantly, the interaction between Time-to-Response and Accuracy was not significant for either stimulation sites (*p* > 0.1) (Figure 2E). This result of double dissociation between FEF and PFv stimulation suggests separation between the processes subserved by the FEF network and those subserved by the PFv network during perceptual decision making. In contrast to the significant effect of Time-to-Response and Accuracy on TMS-EPs in TMS trials, these effects were not significant on the EEG potentials in no-TMS trials (*p* > 0.1). This suggests that the observed modulation of the TMS-EPs cannot be accounted for by the difference in the baseline activity.

**Figure 3:**
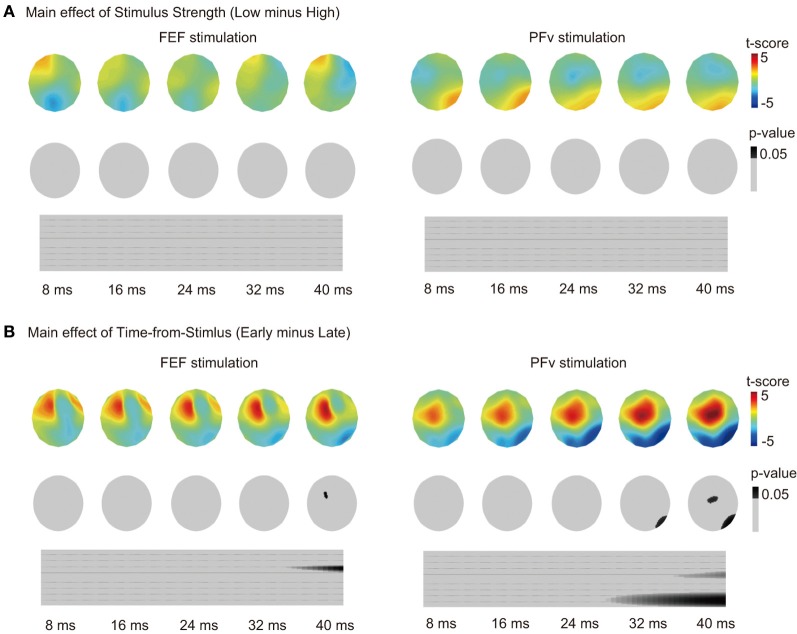
**(A)** Main effect of motion coherence on TMS-EPs. Same format as in Figure 2C. **(B)** Main effect of Time-from-Stimulus on TMS-EPs. Same format as in Figure 2C.

The effect of Accuracy in PFv stimulation experiment can be confounded by the motion coherence level of the stimulus because accuracy changes depending on stimulus strength. However, the effect of motion coherence (categorized as low or high) on the TMS-EPs of PFv stimulation was not significant (*p* > 0.1) (Figure 3A). The effect of motion coherence on the TMS-EPs of FEF stimulation was not significant, either. On the other hand, the effect of Time-to-Response in the FEF stimulation experiment might also reflect the effect of elapsed time from the stimulus onset. When Time-from-Stimulus was entered as a factor instead of Time-to-Response, a significant effect of Time-from-Stimulus on TMS-EP was observed in the FEF stimulation experiment (Figure 3B) although the effect was small and appeared only at a period around 40 ms of TMS. In contrast, a robust effect of the Time-from-Stimulus was observed in the PFv stimulation experiment, despite absence of a significant effect of Time-to-Response in the previous analysis.

### Spread of signals from the FEF and PFv

**Figure 4:**
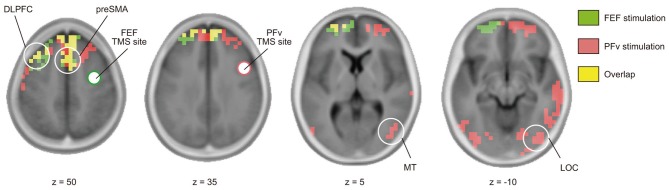
**Effective connectivity based on TMS-EEG.** Distribution of cortical source density estimated from TMS-evoked potentials during 20–40 ms after stimulation of FEF (green) and PFv (red) (threshold: *p* < 0.05, corrected after 5000 permutation). Overlap is shown in yellow. Clusters with significant cortical source density in pre-SMA, left DLPFC (*z* = 50), MT (*z* = 5), and lateral occipital cortex (LOC) (*z* = −10) are indicated by white circle.

Short-latency TMS-EPs have been taken to reflect activation induced by direct neural impulse transmission from the stimulated region. Using cortical source density estimation, we found that both FEF and PFv stimulations induced spread of impulse toward common regions in the left dorsolateral prefrontal cortex (DLPFC) and medial frontal region corresponding to the presupplementary motor area (preSMA) and/or supplementary eye field (SEF) at 20–40 ms of stimulation (Figure 4, Table 2). In addition, PFv stimulation induced activation in posterior visual areas including visual motion-sensitive area MT.

**Table 2:** **Region and MNI coordinate of the cortical source density peak of TMS-EPs**.

**Region**	**Coordinate**
**FEF STIMULATION**	
Rt FPC	5, 65, 20
Rt preSMA	10, 10, 50
Lt DLPFC	−30, 30, 45
**PFv STIMULATION**	
Rt FPC	10, 60, 30
Rt preSMA	5, 10, 60
Lt DLPFC	−35, 20, 50
Lt MT	−55, −75, 0
Rt MT	50, −75, 5
Rt IT	70, −25, −10
Lt LOC	−45, −80, −10
Rt LOC	45, −80, −10

FPC, fronto-polar cortex; IT, inferior temporal cortex; LOC, lateral occipital cortex.

## Discussion

Using the concurrent TMS-EEG technique and applying Random Field Theory to the TMS-EPs data for the first time, we have shown that the connectivity of the FEF network changes depending on the timing relative to behavioral response, whereas the connectivity of the PFv network changes depending on the accuracy of perceptual decision. These results are consistent with our hypothesis that the networks of the FEF and PFv are involved in accumulation and selection of information, respectively. We also obtained results suggesting convergence of signals from the FEF and PFv in medial and lateral prefrontal regions.

### Methodological advantage

We used the TMS-EEG technique to examine task-dependent modulations of neural network connectivity. The idea is that by examining which experimental or behavioral factors modulate the pattern of impulse transmission induced by TMS, we can make inference about the cognitive/computational processes subserved by the network connected with the stimulation site. Compared to fMRI-based effective connectivity analysis such as Granger causality or Dynamic Causal Modeling (Stephan and Roebroeck, 2012), the TMS-EEG technique has three advantages. The first is that by giving TMS on a particular brain region, we are able to examine the state of neural network without a priori assumption about the regions functionally connected to the stimulation site. In fMRI-based effective connectivity analysis, the network connectivity can only be examined within a group of preselected regions, while in TMS-EEG functionally-connected regions can be identified in an exploratory manner.

Secondly, by focusing on short-latency TMS-EPs that occurs in less than 40 ms of the TMS, we can make inference about an early effect of impulse transmission from the stimulated region. It has been shown that inter-regional transmission of neural impulse takes 20–30 ms (Massimini et al., 2005; Morishima et al., 2009; Akaishi et al., 2010; Veniero et al., 2010; Rogasch and Fitzgerald, 2013). Also cortical stimulation and recording studies using subdural electrodes have shown that the induced activation at regions distant from the stimulation site occurs at around 20–30 ms after the stimulation (Matsumoto et al., 2007). It has also been shown that perception of moving phosphene is modulated by TMS on the FEF given 20 ms prior to the MT stimulation (Silvanto et al., 2006). Based on these findings, we consider that the short-latency TMS-EPs that we examined reflect an early effect of the TMS-induced impulse transmission. Our time window of TMS-EP analysis was 20–40 ms after TMS and this seems to be too late if we consider the signal conduction time between bilateral M1s and also between M1 and other regions (SMA, PM, and IPS), which is thought to be around 10 ms or less. This value of 10 ms is based on the results of experiments using two TMS coils (twin coil study): The inter-stimulus interval with which a conditioning TMS pulse has the largest effect on motor-evoked potentials induced by a test TMS pulse is shown to be around 10 ms. By contrast, when the latency of stimulus-induced activation or stimulus-induced modulation of activation is used, the signal conduction time can be estimated to be around 15–30 ms. We consider that the difference is due to the way to measure the effect of stimulation: Changes in cortical excitability as assessed by response to a test pulse TMS can be observed at an earlier timing, whereas the peak of induced response as measured by EEG or ECoG is observed at a later timing. Had we been able to identify the onset of the stimulus-induced response, the signal transmission time based on TME-EPs can be estimated to be shorter.

Thirdly, the TMS-EEG technique allows us to examine the neural network state at a particular time point during cognitive process, that is, at the time point when TMS is given. By varying the timing of TMS relative to an experimental event, we are able to examine dynamic changes in the network connectivity with high temporal resolution. Because of these advantages, there is a possibility that the TMS-EEG technique is more sensitive to the change in the state of neural network than conventional analysis of regional activation. It could be that temporally- dynamic changes in neural activation may not be reflected in the temporally integrated signals such as BOLD signal of fMRI. The benefit of the high temporal resolution in TMS-EEG can be exploited further by appropriate statistical techniques. A large number of data points in the time domain are associated with an increase in false positive results. In the present study, we overcome this problem using Random Field Theory (Worsley et al., 1996).

### Methodological weakness

The TMS-EEG technique has some weakness as well. First of all, the signal induced by TMS is artificial and we do not know if the physiological neural impulses are transmitted across cortical regions in the same way as the TMS-induced signals. Only when the pattern of TMS-induced activation can be shown to be associated with behavior, we can make an argument that the effective connectivity from the stimulated region to the distant region has functional significance and may be associated with physiological mechanism of the network. The generator mechanism for the TMS-induced signals also remains open. Based on the analysis of D- and I-waves (direct and indirect waves) recorded from the hand muscle or spinal cord elicited by M1 stimulation, it is thought that a low-intensity single-pulse TMS initially excites afferent fibers connected to the neurons in the stimulated region (Ziemann and Rothwell, 2000). A large-scale modeling study has shown a more detailed picture for the effect of TMS on local neural circuits within M1 (Esser et al., 2005). First, a TMS pulse directly activates cortical fiber terminals, and induces spiking activity in both excitatory and inhibitory neurons in all cortical layers. The excitatory and inhibitory currents thus induced results in firing of excitatory neurons in layer 5 via synapses made by neurons from layer 2/3. Layer 5 neurons respond to this net depolarization with one to 3 more spikes, which are timed by their intrinsic neuronal properties. It remains open how such a sequence of physiological events changes according to the properties of neural circuits in regions other than M1. It also remains open how this sequence of events interact with the state of circuit at the time of TMS. The cascade of physiological events within the neural circuit elicited by TMS may change depending on the stage of computational processing within the circuit at which a TMS pulse is given. This results in a change in the pattern of TMS-EPs as we have shown in the present study. The underlying neural mechanisms remain open to future studies.

Second point of the weakness of the TME-EEG technique is low S/N ratio. Especially when we examine task-related modulation of the TMS-EPs, the amount of modulation is around 1 μV and we need to average a sufficient number of trials to recover the signal. In the present study, for example, a better way to test the effect of Time-to-Response is to use the real value of the Time-to-Response as a continuous variable and examine the parametric modulation of the TMS-EPs. We were unable to conduct such analysis because of the low S/N ratio of the TMS-EPs, and instead classified trials into binary category of Early and Late. This issue is also related to the limitation of our analysis time windows based on the single unit firing data obtained from monkeys. The timing of neuronal firing in the human brain may differ from that in monkeys, which could have been examined had we been able to examine the TMS-EP data for a narrower time window at multiple time points during the decision process. In addition, we used unusually short pre-stimulus period of 4 ms as the reference for baseline correction. This was to align the amplitude of EEG at the time of TMS at the zero point, but because this duration was arbitrarily chosen this procedure does not guarantee the reliability of obtained results. We need to establish methods for selecting an appropriate pre-TMS epoch for baseline correction and for selecting corresponding epochs of No-TMS trials for comparison, especially when we examine the time varying nature of the TMS-EPs.

Third problem is the ambiguity in localizing the induced activation. Using EEG as a means to record the TMS-induced activation is advantageous in identifying short-latency responses, but spatial localization of the induced activation needs to be analyzed with several assumptions. In the present study, we were unable to identify specific cortical regions in which the TMS-induced activation is modulated by the experimental factors. Concurrent use of TMS-fMRI could be another option to localize the induced activation, which also allows us to examine induced activation in subcortical structures. We, however, lose temporal resolution with fMRI and we are unable to make inference about the efficacy of signal transmission across regions. EEG, on the other hand, allows us to make inference about an early effect of induced signal transmission, but there might be earlier cortico-cortical signal transmission which cannot be detected using the time window of 20–40 ms after TMS. In the stimulated region, the peak of the activation can be observed at 7–9 ms after the onset of the TMS pulse (Ilmoniemi et al., 1997; Rogasch and Fitzgerald, 2013), and it is likely that neural signals are already transmitted to other cortical regions by that time.

### Effect of time-to-response and accumulation of information

We found significant changes in the TMS-EPs in FEF stimulation depending on the timing of TMS relative to subsequent behavioral response (Figure 2C left). Such co-variation with time is consistent with the findings of previous single unit recording studies showing build-up of neural discharge in the FEF, which has been taken to reflect the amount of accumulated sensory evidence used for decision making (Kim and Shadlen, 1999; Ding and Gold, 2012). It is also possible that time-dependent modulation of TMS-EPs of FEF stimulation reflects build-up of motor responses. In contrast to the effect of Time-to-Response, the pattern of TMS-EP after FEF stimulation did not change depending on whether the behavioral response on that trial was correct or error (Figure 2D left), which suggests that the state of FEF neural network reflects the amount of accumulated information regardless of whether subsequent action is based on relevant sensory information or not. Importantly, what we have shown here is the time-dependent modulation in the pattern of signal transmission induced by FEF stimulation, which may reflect changes in the influence from the FEF over other cortical regions. In order to examine temporal dynamics in regional activation using fMRI, the behavioral paradigm has to be set up to allow longer time periods for information accumulation (Ploran et al., 2007). In contrast, the ability of TMS-EEG to examine the network state at a specific time point allows us to test the time-varying nature of the network connectivity such as the network dynamics during fast accumulation processes in decision making.

### Effect of accuracy and selection of task-relevant information

In the PFv stimulation experiment, TMS-EPs changed depending on whether the behavioral response on that trial was correct or error (Figure 2D right). Essential for accurate perceptual decision making is the selection of choice-relevant sensory information. It has been shown that in a visual motion discrimination task, signals from different motion directions are used for decision depending on whether the subject performs a coarse or fine motion discrimination task (Jazayeri and Movshon, 2006). It has also been shown that neurons in the PFv show task-dependent changes in firing rate (Zaksas and Pasternak, 2006; Hussar and Pasternak, 2009), suggesting that these neurons may send task-related selection signals to sensory areas. Our result is consistent with the idea that PFv is involved in selection of choice-relevant information because the network state of the PFv reflects whether the task-relevant sensory information drives the action selection or not in a given trial.

We have also shown that stimulation of the PFv induced activation in the MT (Figure 4), which is consistent with the idea that the PFv sends selection signals to the region involved in processing of task-relevant sensory information. The connection between the PFv and MT has been verified anatomically (Schall et al., 1995). It has also been reported in a fMRI study that a region in the inferior frontal sulcus, which is close to the PFv in the present study, exert causal influence on MT during motion discrimination task for random dot motion with distracting visual features (Kayser et al., 2010).

Additionally, the TMS-EPs after PFv stimulation was not modulated by Time-to-Response (Figure 2C right), but was modulated by Time-from-Stimulus (Figure 3B), which suggests that the state of the PFv network is associated with sensory information processing rather than response generation. The main effect of Time-from-Stimulus, however, can be confounded by the build-up of the subjects' expectancy for a TMS pulse. Such expectancy should exist commonly for FEF stimulation and PFv stimulation conditions, and we indeed found a significant effect of Time-from-Stimulus for both conditions. The TMS-EPs after PFv stimulation, however, did not change depending on stimulus strength (Figure 3A), suggesting that the PFv does not merely represent the externally-provided sensory information.

### Dissociation and convergence between the dorsal and ventral networks

The major finding in the present study is the double dissociation between the FEF and PFv networks. The two networks show significant modulation due to one of the two experimental factors without significant interaction between the two factors (Figures 2C–E). This procedure of testing the effects of two independent factors in a 2 × 2 design is comparable to the conventional analysis of fMRI-based regional activation, but here it is the neural network connectivity that has been examined. Segregation in the functional roles between the FEF and PFv networks, however, does not necessarily exclude the possibility of interaction between them. We in fact found that the two prefrontal networks converge at common regions in the medial and lateral prefrontal cortices (Figure 4), which can be taken to suggest integration of information processed in the FEF and PFv. The TMS-EEG did not show induced activation in all of the anatomically-connected regions after stimulation of FEF and PFv. For example, the FEF and PFv are shown to be anatomically connected with each other (Stanton et al., 1995; Gerbella et al., 2010), but we failed to identify short-latency signal transmission between the two regions. This is probably because the technique allows us to identify only those regions that are functionally connected with the stimulated region in a given task. Since the TMS-EEG technique allows us to make inference about the efficacy of signal transmission from the stimulated region, the regions we have identified, i.e., MT for PFv stimulation and DLPFC and preSMA/SEF for both stimulations, may be regarded as the target regions that receive efferent signals from the FEF and PFv during perceptual decision making.

It is possible that TMS may have stimulated the head skin and induced different patterns of somatosensory-evoked potentials (SEPs) depending on the stimulation site. The distance between the stimulation sites, however, was less than 5 cm. We do not think that such a small difference in somatotopic representation within the head can account for the distinct patterns of TMS-EPs between FEF and PFv stimulation as reported in Figure 2A. Also the activation induced by TMS over FEF and PFv was not observed in somatosensory areas, but rather in regions anatomically connected with the FEF and PFv (Figure 4). We thus consider that the dissociation in the patterns of evoked potentials between the FEF and PFv stimulation reflects the difference in the stimulated cortical regions rather than the difference in stimulated head skin regions. We do accept that the TMS-EPs for each stimulation site may contain a component of SEP, but this would not affect our main conclusion about the dissociation.

In sum, the modulation of the TMS-EPs induced by FEF stimulation by the factor of Time-to-Response is consistent with the idea that the FEF is involved in accumulation of information, whereas the modulation of the TMS-EPs induced by PFv stimulation by the factor of Accuracy is consistent with the idea that the PFv is involved in selection of information. Absence of significant interactions between the two factors for both FEF stimulation and PFv stimulation suggests that the processes of accumulation and selection of information work in parallel during decision making. The overlap of cortical regions in which activation is induced by FEF and PFv stimulations may suggest that the two processes are integrated in medial and lateral prefrontal regions to generate behavioral response. The present study also highlights the feasibility of characterizing the computational processes subserved by a network connected with a particular brain region. What remains open is the mechanism of the modulation of neural network connectivity. It has been suggested that low-intensity TMS as we used in the present study primarily activates the afferent fibers that are connected to the neurons in the stimulated region, which then activates those neurons that project to other regions (Esser et al., 2005). Modulation of the TMS-EPs may thus reflect changes in the balance between the excitatory and inhibitory activity of neurons within the stimulated region (Silvanto et al., 2008; Pasley et al., 2009). Another possibility is that the modulation occurs at the regions that receive signals from the stimulated region. More specifically it may be due to the change in the efficacy of synaptic transmission within the target regions that receive inputs from the stimulated region. In either case, the modulation of TMS-EPs can be taken to reflect the state of neural network and its association with experimental or behavioral factors allows us to make inference about the computational processes performed in the neural network connected with a particular region.

## Author contributions

Rei Akaishi planned, designed and conducted the experiments. Naoko Ueda helped conducting experiments. Rei Akaishi and Katsuyuki Sakai analyzed the data and wrote the paper.

### Conflict of interest statement

The authors declare that the research was conducted in the absence of any commercial or financial relationships that could be construed as a potential conflict of interest.
